# Building block 3D printing based on molecular self-assembly monolayer with self-healing properties

**DOI:** 10.1038/s41598-022-10875-9

**Published:** 2022-04-26

**Authors:** Hicham Hamoudi, Golibjon R. Berdiyorov, Atef Zekri, Yongfeng Tong, Said Mansour, Vladimir A. Esaulov, Kamal Youcef-Toumi

**Affiliations:** 1 Qatar Environment and Energy Research Institute, Hamad Bin Khalifa University, P.O. BOX 34110, Doha, Qatar; 2 Institut Des Sciences Moléculaires d’Orsay, UMR 8214 CNRS-Université, bât 520, Université Paris Sud, Université Paris Saclay, 91405 Orsay, France; 3 Mechatronics Research Laboratory, Massachusetts Institute of Technology, 77 Massachusetts Ave, Cambridge, MA 02139 USA

**Keywords:** Nanoscale materials, Soft materials, Techniques and instrumentation

## Abstract

The spontaneous formation of biological substances, such as human organs, are governed by different stimuli driven by complex 3D self-organization protocols at the molecular level. The fundamentals of such molecular self-assembly processes are critical for fabrication of advanced technological components in nature. We propose and experimentally demonstrate a promising 3D printing method with self-healing property based on molecular self-assembly-monolayer principles, which is conceptually different than the existing 3D printing protocols. The proposed molecular building-block approach uses metal ion-mediated continuous self-assembly of organic molecular at liquid–liquid interfaces to create 2D and 3D structures. Using this technique, we directly printed nanosheets and 3D rods using dithiol molecules as building block units.

## Introduction

What is the origin of the high efficiency and self-healing ability of biological devices? One of the most efficient machines in nature is flagellum, which is responsible for the motion of different microorganism such as bacteria^1^. This nano-machine consists of dozens of self-organized proteins. Human brain is also a product of 3D self-assembly process with nervous cells as building blocks^2,3^. The fabrication of such efficient technorganic devices requires a very advanced 3D printing machine, which can control the matter growth at the molecular level.

In general, 3D printing refers to a broad range of digitally controlled additive manufacturing processes for creation of complex 3D objects and considered as a disruptive approach for conventional materials fabrication^4,5^. There are several well-stablished 3D printing technologies such as inkjet printing, stereolithography, selective laser sintering and fused deposition manufacturing^6^. Despite computer generated processing, it is not straightforward to control the resolution of 3D printed structures due to the limitations of the printing methods. On the other hand, the control of the structural properties of the resulting 3D objects at the molecular level would be very important in many different technology areas. For example, such molecular-level printing technology can act as a disruptive force for today's traditional lithographic technology.

Here, we report molecular self-assembly-based 3D printing method based on self-assembled monolayer (SAM) approach^7^, which is capable of fast and continuous printing of different 3D objects with self-healing properties using molecules as building blocks and metal ions as mediators. The approach uses advances of liquid–liquid interface engineering to enable continuous formation of SAMs at the interface via intermolecular interactions. The present 3D printing approach has several advantages such as defect-free materials creation and room-temperature processing. Moreover, the resulting 3D structures show self-healing properties which are characteristic for living tissues in repairing minor damages.

Creating self-healing structures is one of the main objectives of additive manufacturing as irreversible failures limit the performance of 3D printed objects. In polymeric materials, chemical reactions such as covalent bonding, ionic and electrostatic interactions, and supramolecular assemblies, are the primary mechanisms that lead to self-healing^8^. In the present method, the self-healing occurs due to the formation of dynamic metal-sulfur covalent bond formation at the liquid–liquid interface without any external trigger. This self-healing process enables one to create complex 3D structures from molecular building blocks with specific edge groups.

**Figure 1:**
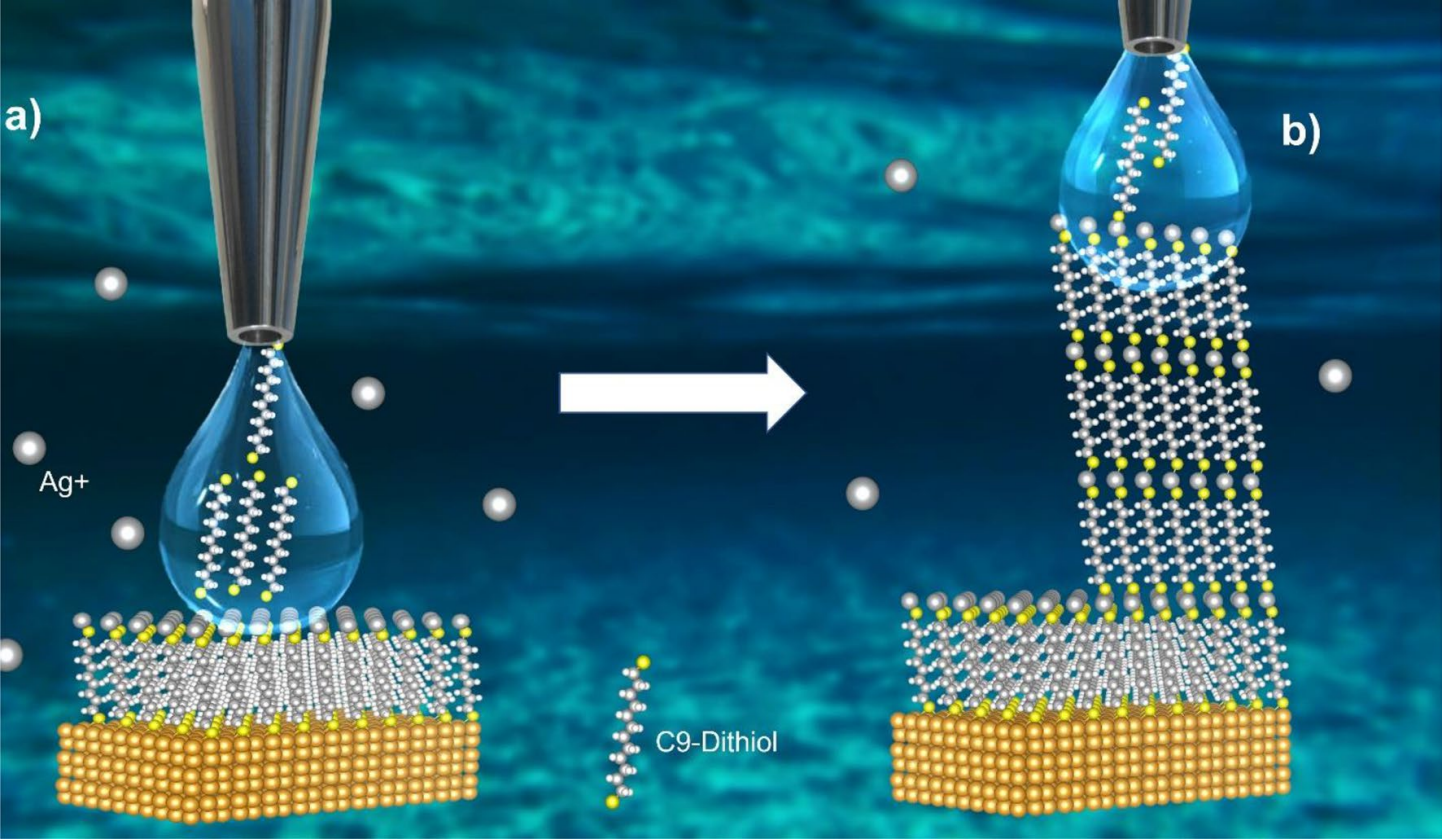
Principle of molecular self-assembly based 3D printing. Schematics of continuous self-assembly of C9-dithiol molecules at water-hexane interface mediated by silver atoms. (**a**) Solution containing dithiol molecules injected through a contact angle nozzle on top of the metal-SAM interface. (**b**) Injection of the dithiol molecule solution and pulling up the nozzle at the same time enable to create a periodic and continuous metals-SAMs multilayer structures.

The general principle of the present molecular building-block 3D printing approach is illustrated in Fig. 1. First, a high-quality dithiol SAM on metallic substrate will be prepared using well-established protocols to avoid oxidation of the sulfurs-end groups^9–11^. Dithiol molecules are the best building blocks for the present 3D printing approach due to the possibility of grafting metal atoms required for continuous self-assembly. The resulting SAM will be immersed into water containing metal atom precursor. Due to chemical reactions, the surface of the SAM will be grafted with the metal atoms (see Fig. 1a). In the second stage, another solution containing dithiol molecules will be injected through a contact angle nozzle on top of the metal-SAM structure (see Fig. 1a). Some of the molecules in this solution will form the second layer of SAM due to the interaction with silver metal atoms adsorbed on top of the previous SAM and the remaining of the molecules will be expelled up together with the solution. The surface of the newly formed SAM will also be covered by metal atoms instantaneously. By the injection of the dithiol molecule solution and pulling up the nozzle at the same time will enable us to create a periodic and continuous metals-SAMs multilayer structures made by the molecular-SAM building block method as illustrated in Fig. 1b.

To demonstrate the working principle of the proposed method, we conduct proof-of-concept experiments using nonane-dithiol (C9) molecules as building blocks solvated either in n-hexane or in ethanol. The structures are created in water containing silver atom precursor (silver nitrate) using the experimental setup as described in the supplementary materials (see Fig. S1). The liquids containing the molecules are injected with flow speed of 650 µl/min and vertical nozzle speed of around 0.2 mm/s.

## Methods

### Sample preparation

All the chemicals and solvents (nonanedithiol, ethanol (99%) and hexane) were purchased from Sigma Aldrich and used for sample preparation without further purification. Pure ethanol and hexane were degassed under nitrogen gas flow for 20 min. Au (111) on Si was rinsed with absolute ethanol 3 times and dried under nitrogen gas flow prior to use. The 10 mM solution of C9 dithiol is prepared by dissolving the molecules in the pre-degassed hexane. The clean substrate is then immersed into the solution for SAMs formation at room temperature for C9 dithiol. The substrates are kept for 1 h in dark condition to avoid light-induced molecular oxidation. The resulting SAMs are then sufficiently rinsed with hexane and dried under N2 flow. The C9-dith/Au sample is then placed in the bottle filled with 100 mM of AgNO_3_ water solution, which is pre-degassed for 30 min to avoid oxidation. A glass Syringe is utilized to store the well-degassed dithiol solution previously prepared and installed onto a contact angle system. The injection of the solution is accomplished by controlling the dissipation of solution flow through the contact angle nozzle.

### Photoemission measurements

The photoemission measurements were performed on a standard Thermo Fisher ESCALAB 250XI type X-ray photoelectron spectroscopy (XSP) platform. A monochromatic Al Kα Anode X-ray beam of 1486.6 eV was used with an energy resolution of 0.5 eV. The XPS spectra were obtained with a normal emission and a beam incident of 45° to the surface normal. All the energy values were calibrated with respect to the Au4f. located at 84 eV.

Scanning electron microscopy (SEM) measurements were performed on an FEI Versa3D instrument (Hillsboro, OR, USA) at an acceleration voltages 7 kV, 15 kV and 20 kV, and at working distance of ~10 mm. For EDS, a Bruker Quantax 800 detector (Billerica, MA, USA) was used at a fixed 7 kV acceleration voltage.

### Transmission electron microscopy measurements

Transmission electron microscopy (TEM) and STEM-EDS were performed on a TALOS (FEI, Hillsboro, Oregon, USA) field emission gun transmission electron microscope equipped with an FEI EDS detector and a high angle annular dark-field (HAADF) detector operating at 200 kV. For qualitative elemental chemical analysis, ESPRIT software from Bruker was used. Conventional TEM bright field images and STEM HAADF (Z-contrast) images were taken at different regions along the nanosheet in order to obtain the average particle size as well as the particle size distribution of Ag in the matrix (silver sulfur and carbon matrix). By analyzing different areas of the film across its thickness, the Feret diameters of several particles were calculated based on STEM-HAADF imaging. ImageJ and Digital Micrograph software were then used for particle size analysis as well as the particle shape analysis (circularity). The quantitative statistical analysis of the images is performed in order to characterize the particle size (Feret diameter) (d), standard deviation in size (σ), circularity (c).

## Results and discussions

### 2D nanosheet formation at n-hexane-water interface

**Figure 2:**
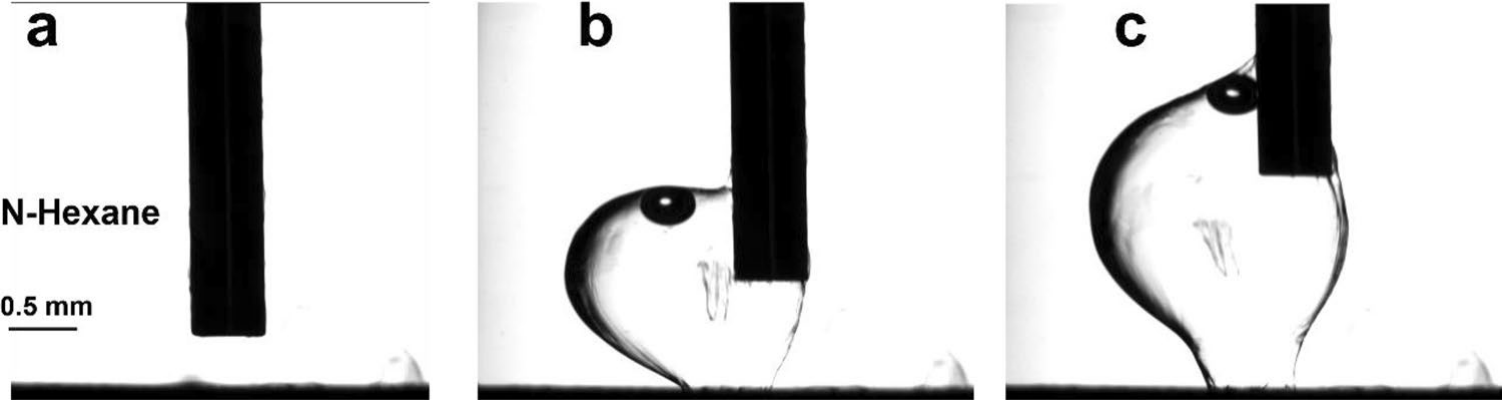
3D printed objects: case 1. Optical images of the formation of 2D nanosheets from C9 molecules at water-hexane interface. (**a**) Approaching the nozzle to the substrate, (**b**) injection of the C9-dithiol in hexane solution, (**c**) injection of the C9-dithiol in hexane solution and pulling up the nozzle at the same time.

Figure 2 shows the optical images taken during the formation of structures at water-hexane interface. We first bring the nozzle at a distance close to the SAM-surface (Fig. 2a) and then start expelling the n-hexane solution containing C9 molecules continuously and move the nozzle vertically up. Due to the interaction with silver atoms adsorbed on top of the first the SAM, the C9 molecules will form the second monolayer. However, because of the large difference in the polarity of water and hexane, which prevents mixing of hexane with water, metal atoms cannot fully penetrate the injected hexane solution and therefore the molecules will be decorated with metal atoms only at the water-hexane interface. Consequently, we form 2D nanosheets with a finite thickness (Fig. 2b). As seen in Fig. 2c, the nanosheet produced at the interface between water and n-hexane take the form of a bubble separating the two liquids. Consequently, a very thin nanosheet separating water and hexane formed during the process. Moving the nozzle upward and simultaneously injecting the second liquid enables further growth of the nanosheet with hexane (which comes from the nozzle) confined inside the bubble. Supplementary online video 1 shows the process of the formation of the 2D nanosheet using n-hexane. The object remains on the substrate once the nozzle is moved vertically up without injection. By fishing (removing the printed object from the substrate) the resulting nanosheet, the encapsulated hexane evaporates, and the formed nanosheet remains deposited on the substrate. To make qualitative evaluation of mechanical stability of the 3D printed structures, we have conducted an experiment as demonstrated in Supplementary online video 2. It is seen from this video that the resulting 3D objects are mechanically stable during the tensile test.

### The case of ethanol solvent-3D structure formation

**Figure 3:**
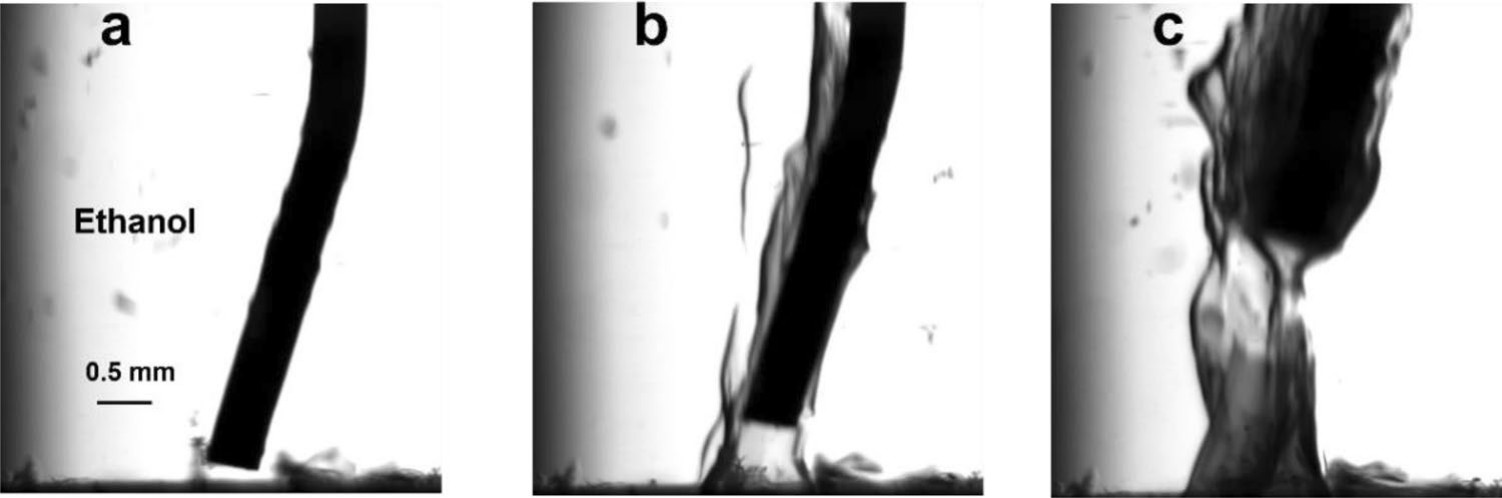
3D printed objects: case 2. Optical images of the formation of 3D structure from C9 molecules at the water–ethanol interface. (**a**) The nozzle is approaching to the substrate, (**b**) injection of the C9-dithiol in ethanol solution, (**c**) injection of the C9-dithiol in ethanol solution and pulling up the nozzle at the same time.

Figure 3 shows one of the 3D structures created using ethanol as a solvent for the C9 molecules. Clear structural difference is seen between the objects created in this case as compared to the samples created using n-hexane (see Fig. 2). Namely, we do not have bubble structures as in the former case, but rather obtain solid objects (Fig. 3b,c) with the lateral size determined by the size of the nozzle (for a given location of the nozzle). The latter is due to the fact that ethanol has a similar polarity as water which allows full penetration of the metal ions to decorate the surface of the self-assembled molecules. Supplementary video 3 shows the process of the formation of the 3D carbon structure on top of the substrate using ethanol. As in the former case, the 3D object remains on the metallic surface once the nozzle is removed from the printing area. The resulting 3D object can be transferred to another substrate for further analysis.

### Characteristics of the printed structures

**Figure 4:**
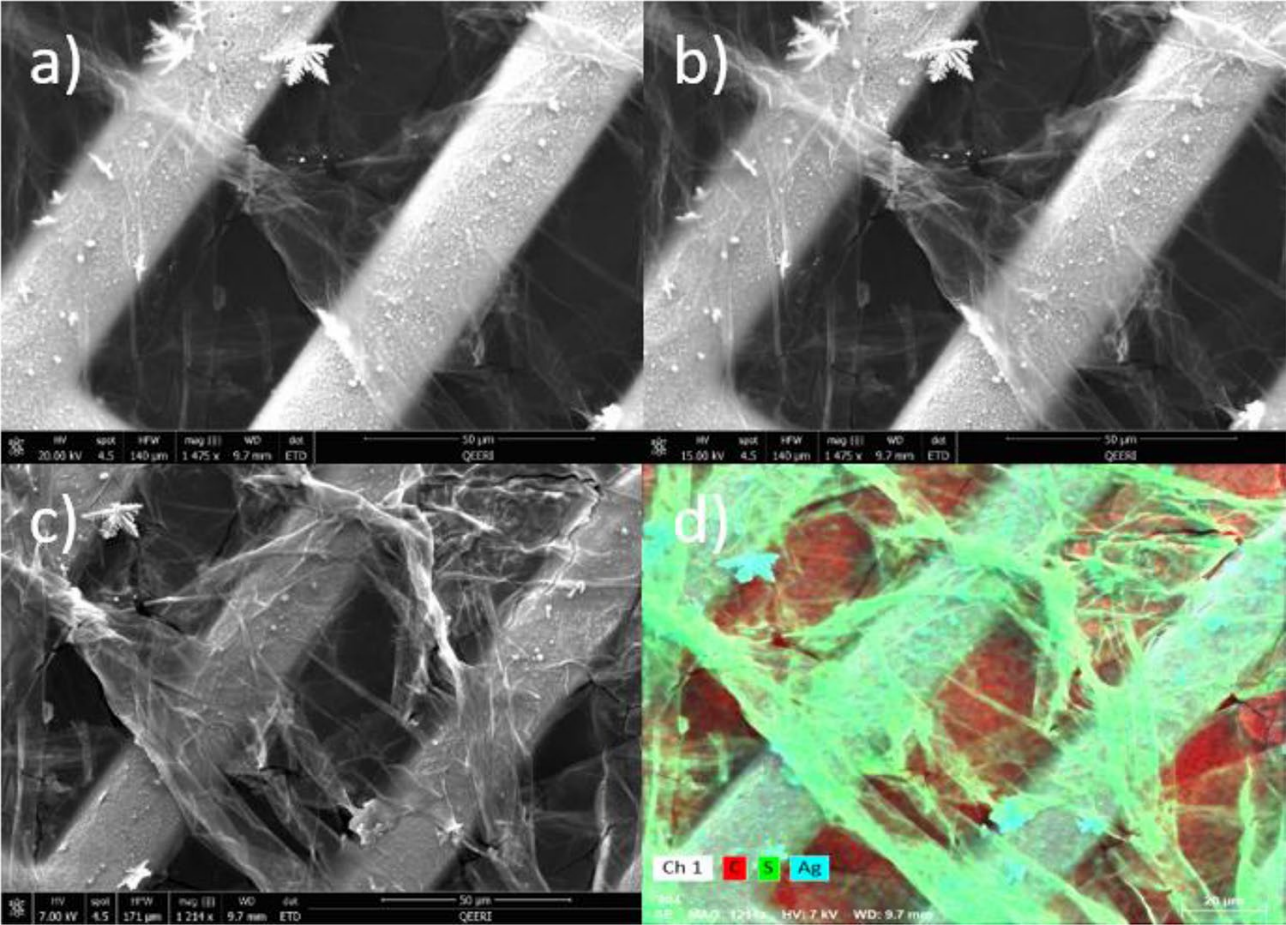
The morphology of the thin films produced using n-hexane. Scanning electron microscopy images taken at accelerating voltages (**a**) 20 kV (**b**) 15 kV and (**c**) 7 kV. (**d**) Elemental distribution of sulfur (green), carbon (red) and silver (cyan).

We have characterized the structural and chemical properties of the resulting 2D nanosheets (case of n-hexane) and 3D objects (case of ethanol) using SEM, TEM, and XPS measurements. Figure 4a–c show the SEM images of the structures created using n-hexane solvent at different e-beam accelerating voltages. The thin nanosheet structure of the obtained material is clearly seen in Fig. 4a. By decreasing the voltage electron beam, the nanosheet morphology becomes more visible (see Fig. 4b,c) due to the decrease of the interaction volume between the incident beam and the nanosheet. This allows a higher surface sensitivity during imaging. Figure 4d shows the elemental composition of the material with carbon presented in red and sulfur in green. Note that pronounced carbon signal between the TEM-grids is because the grid was fixed on a carbon tape.

**Figure 5:**
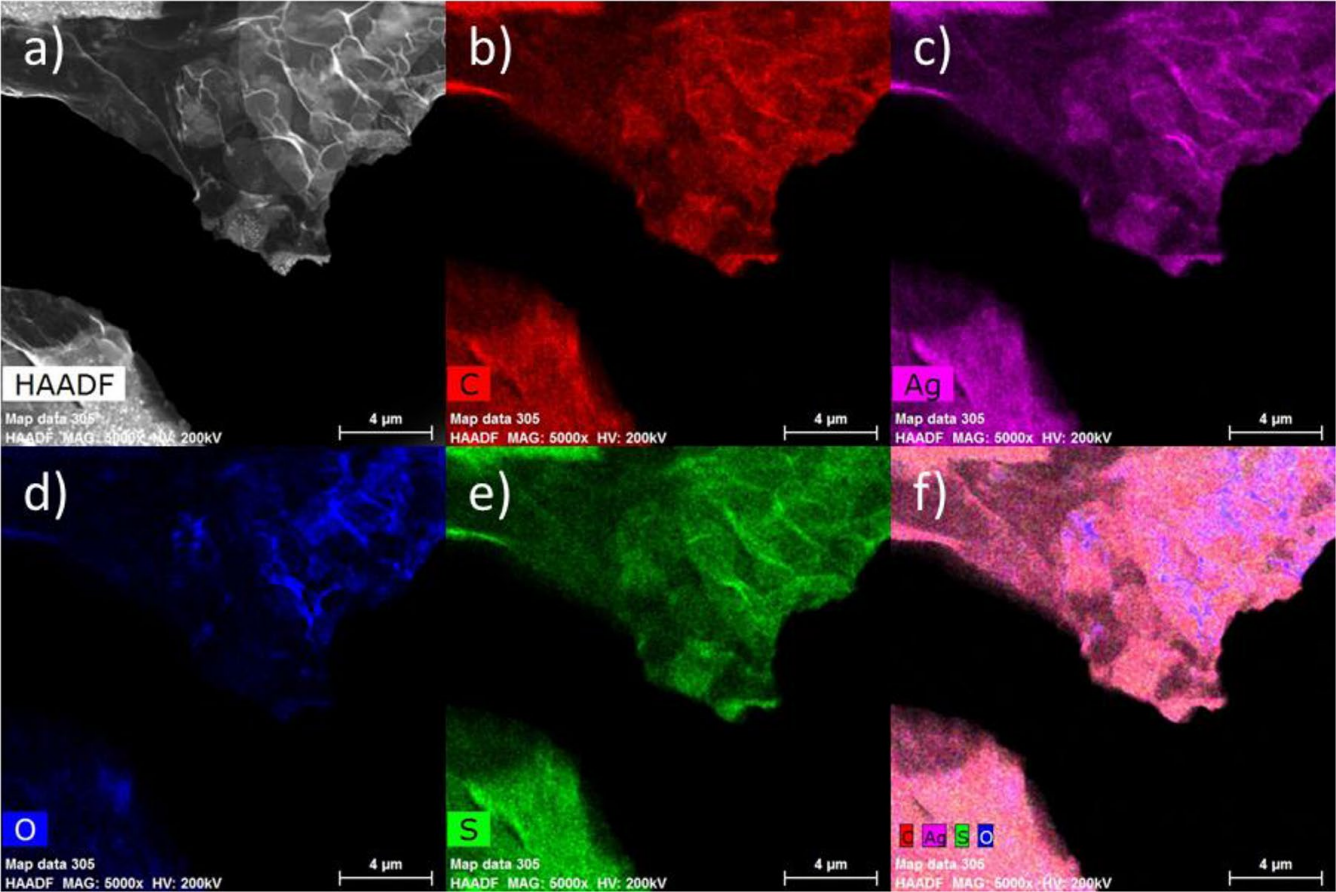
Elemental composition of the nanosheet structure created using n-hexane. (**a**) STM HAADF images of the nanosheet deposited on TEM grid. (b-e) Distribution of carbon (**b**), silver (**c**), oxygen (**d**) and sulfur (**e**). (**f**) the overlap mapping of the four elements (carbon, silver, sulfur, and oxygen).

Chemical properties of the resulting carbon materials are studied using TEM at different imaging modes (HR-TEM as well as STEM). It is well known that in many cases electron beams can induce significant structural changes and radiation damage to carbon materials^12,13^, which would prevent obtaining the desired information in imaging and chemical analysis in the TEM. However, the formed carbon structures in this study show significant resistance to the electron beam indicating good stability of the samples. Figure 5a–f show the STEM HAADF (Z-contrast) images of the nanosheet deposited on TEM grid. The nanosheet structure of the obtained materials is clearly visible in Fig. 5a. The elemental mapping in Fig. 5b–f confirms the coexistence of the composing elements; carbon (red), silver (pink) and sulfur (green), which are homogeneously distributed (as seen in Fig. 5f) in the nanosheet. The quantitative composition is at.% C = 69.80, at.% S = 10.63, at.% Ag = 14.44 and O = 0.33 at.%. The presence of oxygen in the structures is due to sample exposure to open air during the transfer from the 3D printer to the TEM.

**Figure 6:**
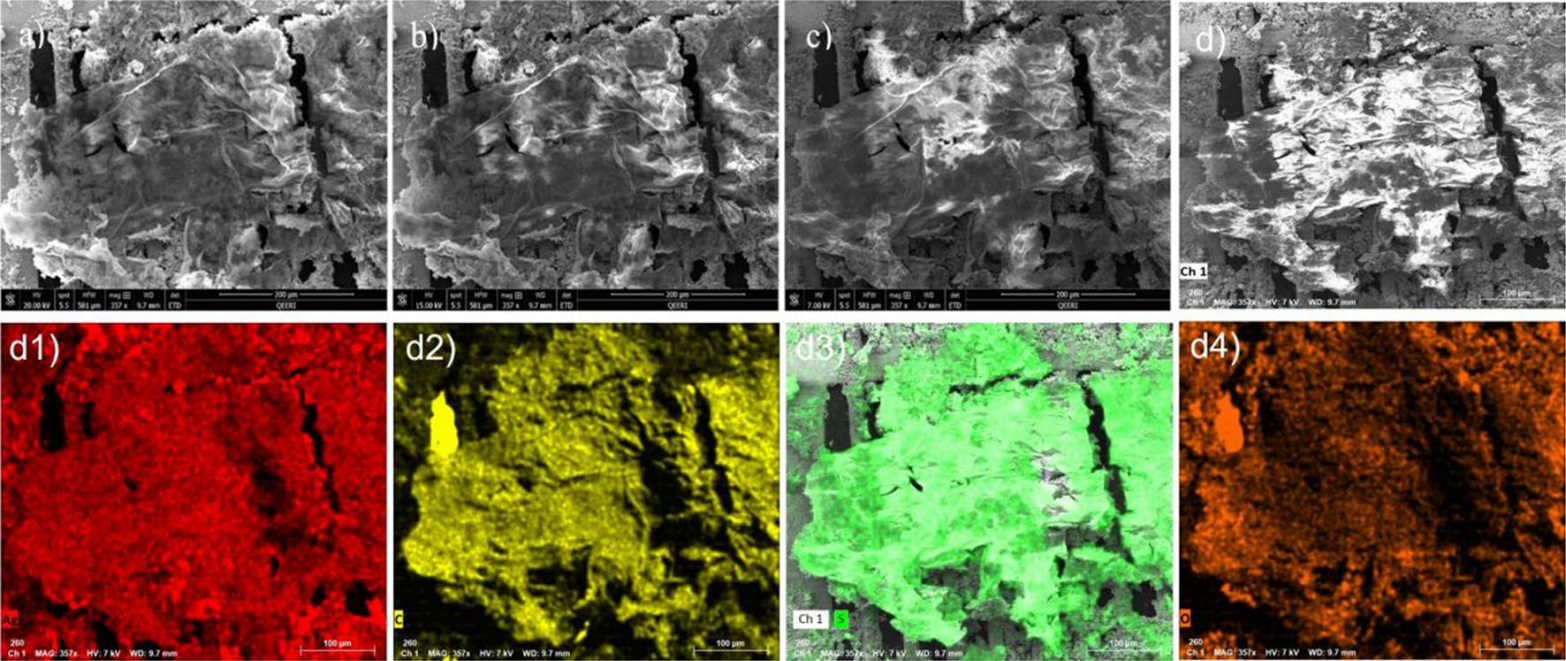
The morphology of the 3D structures created using ethanol. SEM images are taken at different e-beam accelerating voltages at: 7 (**a**), 15 (**b**), and 20 kV (**c**). (**d**) Elemental distribution of: (**d1**) silver, (**d2**) carbon, (**d3**) sulfur, and (**d4**) oxygen.

Figure 6 shows the SEM images and elemental distribution of the 3D objects created using ethanol solvent at different accelerating voltages. Although decreasing the accelerating voltage down to 7 kV, the material morphology seems to be the same as using 20 kV, which indicates a larger thickness of the material as compared to nanosheet structure presented in Fig. 4.

**Figure 7:**
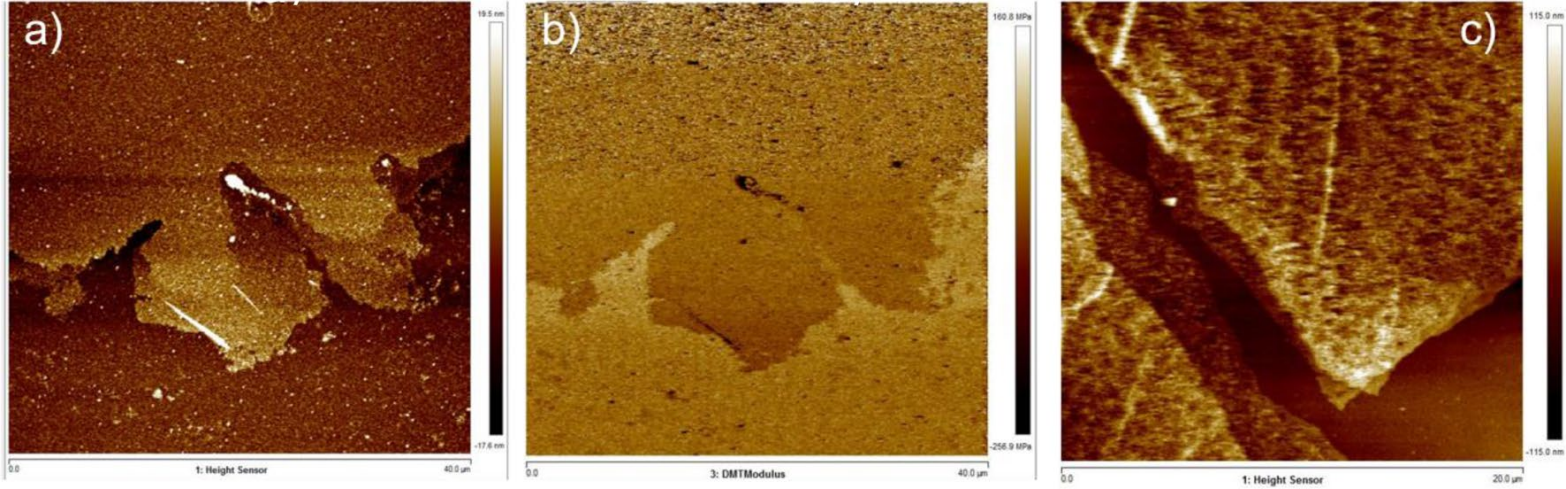
AFM images of the 3D structures created using n-hexane. (**a**) height image and (**b**) DMT module mapping (**c**) AFM images of the 3D structures created using n-hexane solvent after evaporation of encapsulated hexane.

To get more accurate information about the thickness of the created materials, we have conducted peak-force atomic force microscope (PF-AFM) measurements. For the nanosheet structures created using the n-hexane, we can see in AFM height image (Fig. 7a) an overlay of multiple nanosheet layers. The nanosheet was placed on silicon substrate, to clearly distinguish between both phases through the color gradient on the DMT Modulus mapping as shown in Fig. 7b. The estimated thickness of the nanosheet structure created using n-hexane was ~ 5 nm (see Fig. S2). The AFM imaging in Fig. 7c clearly shows that both nanosheet sides formed from a single bubble in n-hexane overlap on each other due to the evaporation of hexane in air. On the contrary, the thickness of the structures created using ethanol was varying between 250 nm up to 4 µm as revealed in the profilometer measurements (see Fig. S3).

**Figure 8:**
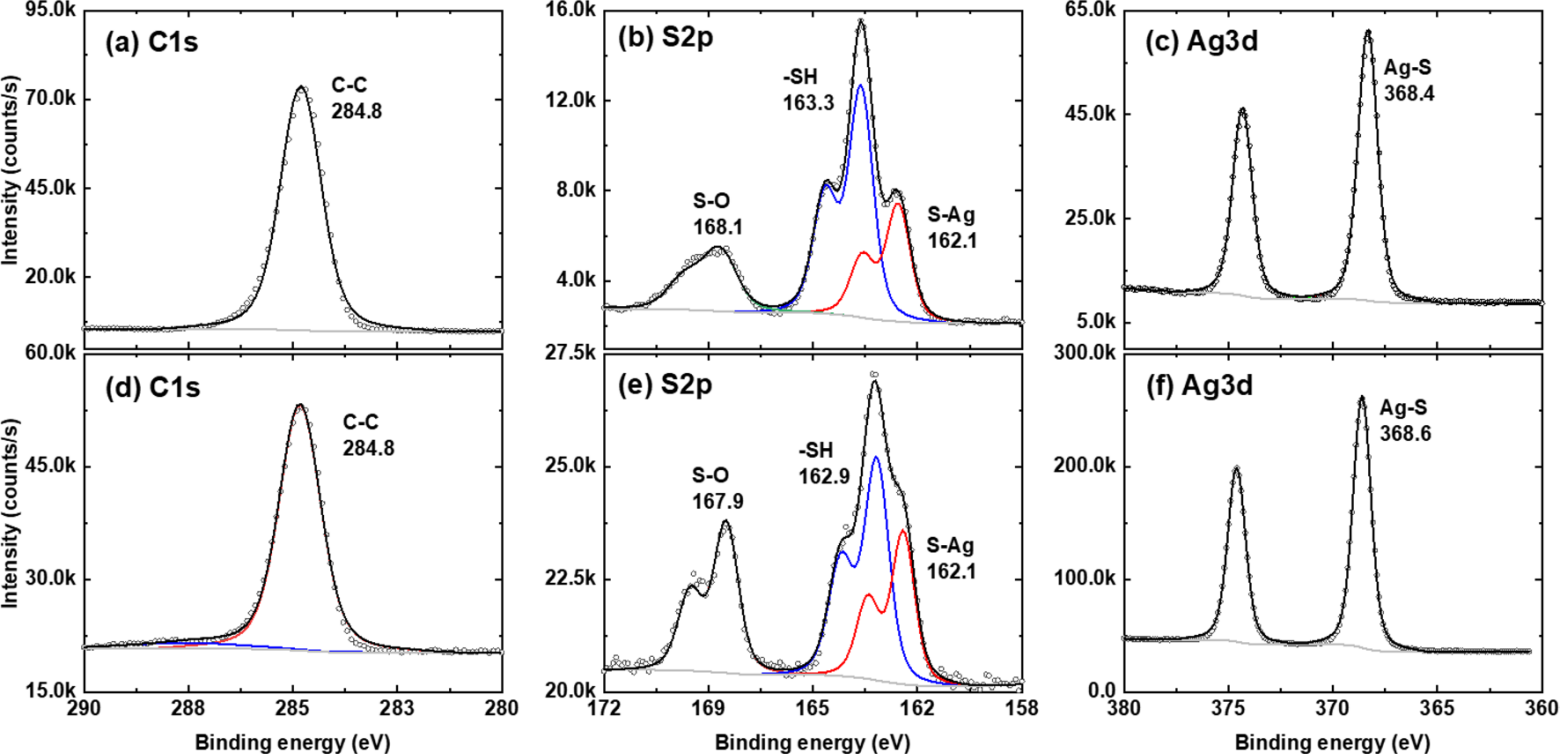
XPS chemical analysis. C1s (**a**,**d**), S2p (**b**,**e**) and Ag3d (**c**,**f**) core level XPS spectra of the 3D printed structures using n-hexane (**a**–**c**) ethanol (**d**–**f**).

We have also studied the properties of the 3D printed structures using XPS measurements. Figure 8 shows the C 1s, S 2p and Ag 3d core level spectra of the samples created using n-hexane (a–c) and ethanol (d–f). Both samples show qualitatively similar XPS spectra with different signals intensities. Small difference is obtained for S–O signal in the spectra (at 168 eV) because of the nature of the solvent^9^. Figure S6 in the supplementary materials show the XPS spectra of a monolayer of C9 dithiol molecules on Au (111) substrate before starting the 3D printing process.

### Effect of annealing and metal nanoparticle formation

**Figure 9:**
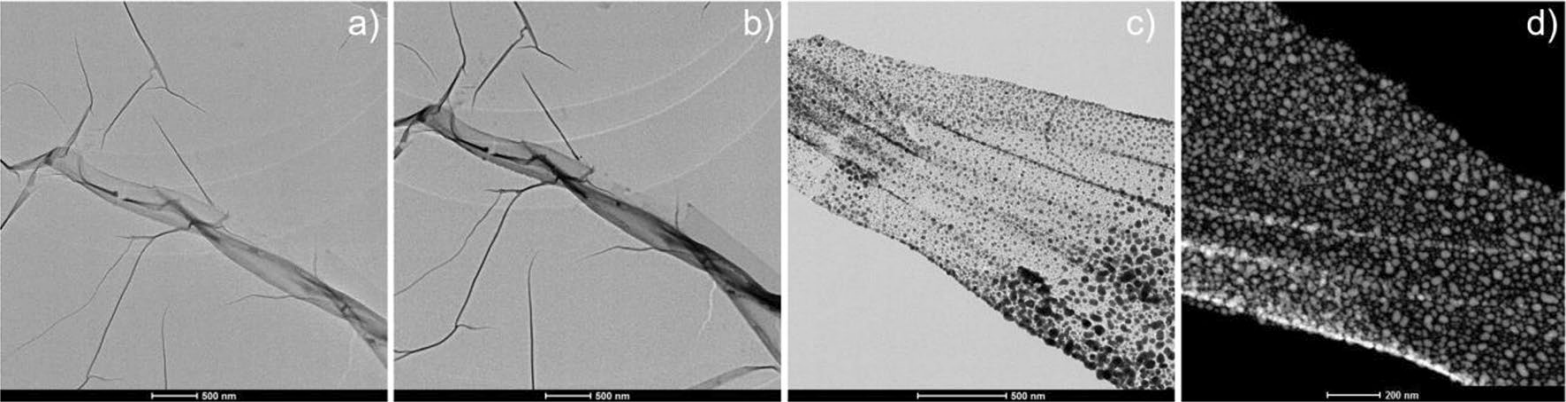
Thermal effect on the 2D printed structures. TEM micrographs of the nano sheet during the In-situ annealing: (**a**) at 60 °C, (**b**) at 200 °C, (**c**) at 400 C, (**d**) STEM HAADF at 550 °C.

Finally, we analyzed thermal effect on the 3D printed structures. Figure 9 shows the TEM images of the nanosheet material created using n-hexane at different in-situ heating temperatures. The annealing treatment at temperatures up to 400 °C results in the formation of Ag nanoparticles throughout the carbon-host matrix, with a temperature-dependent size distribution. The growth of Ag nanoparticles was studied by in-situ annealing process using electron diffraction. The diffraction study (Fig. S4) shows that the temperature-trigger of the formation of the nanoparticles is between 100 and 200 °C, the analysis of hundreds of particles in the film showed an average diameter of 3.03 nm at 200 °C (Fig. S5). Subsequent annealing at 550 °C produces aggregation of particles forming a larger Ag-nanoparticle, with particle size of more than 60 nm, which can be attributed to the recrystallization and grain growth process^11^.

**Figure 10:**
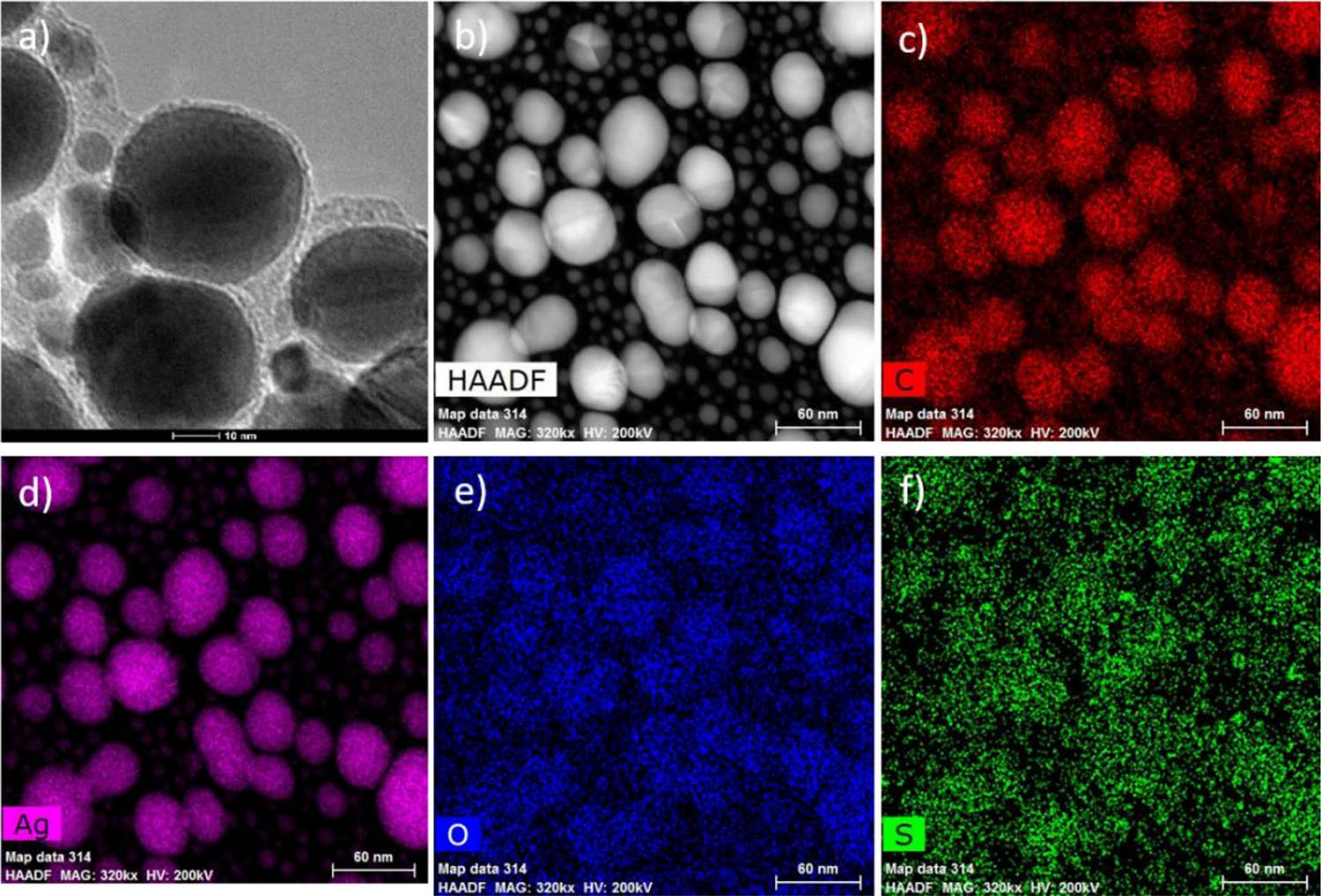
Thermal annealing induced nanoparticles formation. (**a**,**b**) STEM/TEM micrograph of the Ag nanoparticles at 550 °C on the TEM grid, in addition to elemental maps of (**c**) Carbon on red (**d**) silver on pink (**e**) Oxygen on blue (**f**) Sulfur on green and surrounding matrix. The results show homogenized distributions of S on the nano sheet.

We evaluate the average size of the nanoparticles and their distribution in the carbon-matrix at different annealing temperatures. Figure 10a,b show the enlarged images of Ag nanoparticles on the carbon nanosheet on the TEM grid. Ag nanoparticles of different sizes are created during the annealing process. Figure 10b–f show the elemental compositions of the material. It is seen from this figure that Ag nanoparticles decorated homogenously by C and S atoms (see Fig. 10a,f).

Another important feature that affects the particles size is obviously the chemical nature of the surrounding matrix. The rate of diffusion of some metals such as Ag throughout a self-assembly building block 3D depends significantly on the Ag–S binding energies. Therefore, the host matrix is expected to have an important role on the nucleation and growth mechanisms of the Ag nanoparticles. As we can see in Fig. 10a, some carbon-based phase is still present around the nanoparticles^11^. This matrix plays an important role on stabilizing the nanoparticle growth by imposing a pressure on the particle surface.

### Self-healing properties of the 3D printed objects

To test the self-healing properties of the 3D-printed objects, two separately printed structures are placed in contact as demonstrated in Supplementary video 4. Both structures are created using n-hexane as a solvent for the molecules. It is seen that the fusion between the two structures does not happen in this case because the molecular sulfur-end group of both structures are saturated by silver ions, resulting in electrostatic repulsion between the objects. Next, as demonstrated in Supplementary video 5, we inject additional molecules through hexane solution to decorate the surface of the top object with unsaturated molecules. The merging occurs instantly, and the objects are self-repaired. This adhesion process is attributed to the formation of silver-sulfur bonds. Additional experiments show that self-healing can be obtained in freshly printed specimens as well as in prior-printed objects with the same efficiency and speed.

**Figure 11:**
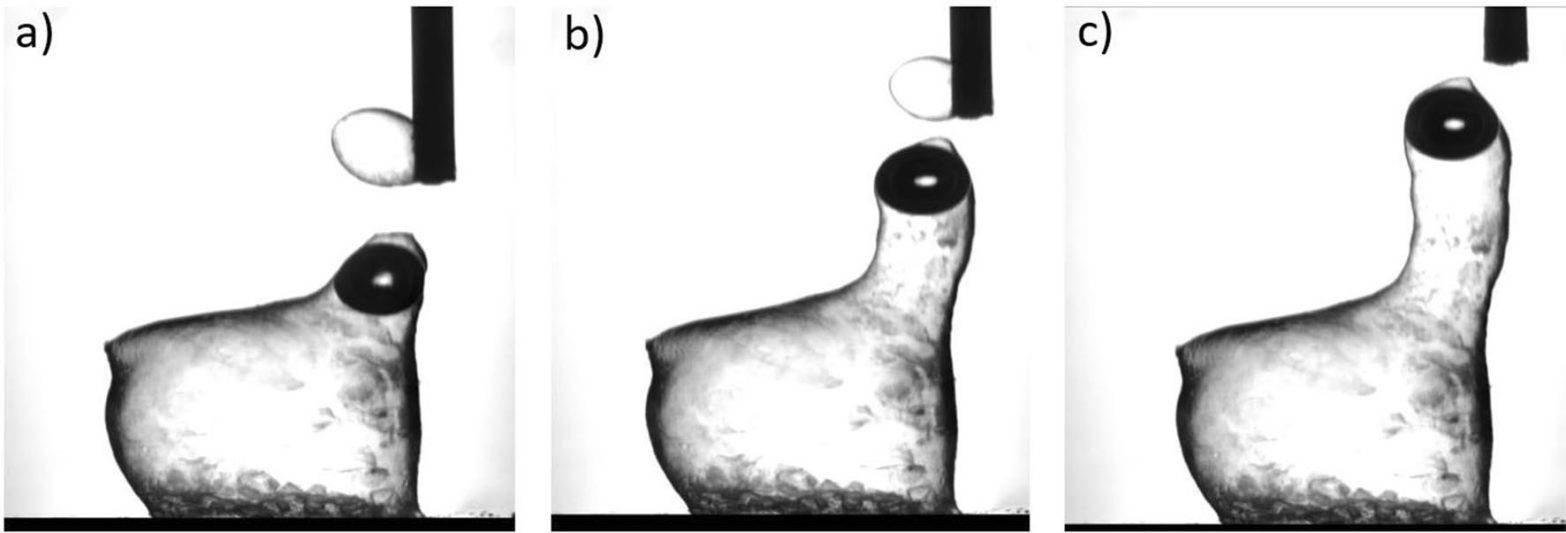
Self-healing of 3D printed objects. Optical images of the formation of tubular structure after self-healing. (**a**) A nozzle with a 3D printed object is approaching a 3D printed structure on the substrate. The dark spot is the air bubble inside the object. (**b**) Hexane solution is injected when these to objects are in contact. (**c**) When these to objects are merged due to the self-healing mechanism the nozzle is moved vertically up without injecting the solvent. The air bubble moves up during the processes confirming the tubular structure of the object after self-healing.

As we have shown in Fig. 2, using n-hexane as a solvent result in tubular structures. To confirm that the objects after self-healing preserve the tubular structure, we have conducted the following experiment. We first created a tubular structure and injected an air bubble inside the tube (see dark sport in Fig. 11a). After that we made a direct contact between this sample and another tubular structure and started injecting additional molecules. The two objects rejoin together, and the air bubble starts moving up (Fig. 11b). This indicates that the tubular structure of the resulting object is preserved after the printing completion (Fig. 11c). The complete self-healing process is shown in Supplementary video 6.

## Conclusions

We have experimentally demonstrated a molecular 3D printing method to create different 3D objects with self-healing properties. The present method is based on continuous self-assembly of organic molecules at liquid–liquid interfaces mediated by metal ions in the solution. Proof-of-concept experiments are conducted using C9 dithiol molecules and structural and thermal properties of the 3D printed objects are studied using different characterization tools. The present molecular building block 3D approach is conceptually different than the present-day 3D printing protocols and can open promising perspectives in material discovery and creating nano-technorganic materials with functionalities out of the scope of traditional nano-fabrication methods. For example, complex metal–organic hybrid structures can be printed as shown in Supplementary video 7. Self-repairing of the 3D printed objects can be obtained at room temperature without any external trigger which enables creation of more complex structures. However, several challenges remain to be overcome such as controlling printing process at the molecular level.

## Supplementary Information

Supplementary Information 1.

Supplementary Video 1.

Supplementary Video 2.

Supplementary Video 3.

Supplementary Video 4.

Supplementary Video 5.

Supplementary Video 6.

Supplementary Video 7.

## Data Availability

All data are available in the main text or the supplementary materials. Additional data are available by the corresponding author upon request.
