# Supplementary material for: Building block 3D printing based on molecular self-assembly monolayer with self-healing properties
Source: Sci Rep. 2022 Apr 26;12:6806. doi: 10.1038/s41598-022-10875-9 (PMC9043216; doi:10.1038/s41598-022-10875-9)
Supplement: Supplementary file 1 — Supplementary Information 1. [file 41598_2022_10875_MOESM1_ESM.docx]

Supplementary Materials for

**Building block 3D printing based on Molecular self-assembly monolayer with self-healing properties**

*Hicham Hamoudi, Golibjon R. Berdiyorov, Atef Zekri, Yongfeng Tong, Said Mansour, Vladimir A. Esaulov, Kamal Youcef-Toumi*

Correspondence to: [hhamoudi@hbku.edu.qa](mailto:hhamoudi@hbku.edu.qa), hichamhamoudia@gmail.com

**This PDF file includes:**

Materials and Methods

Figs. S1 to S5

Captions for Movies V1 to V7

**Other Supplementary Materials for this manuscript include the following:**

Movies V1 to V7

Experimental setup


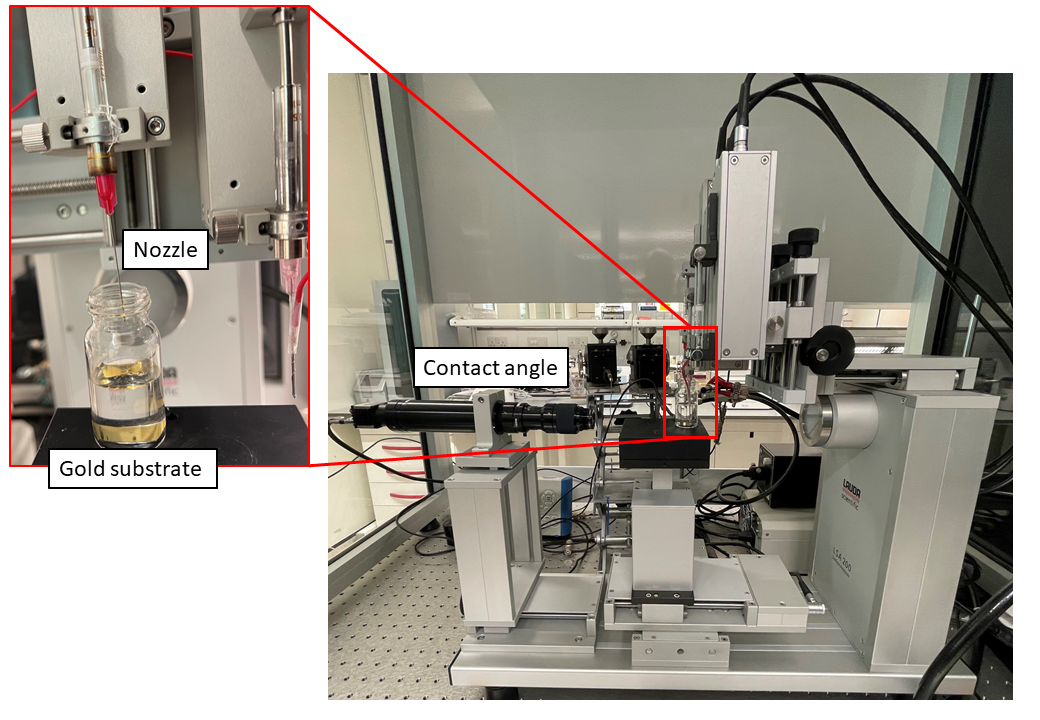

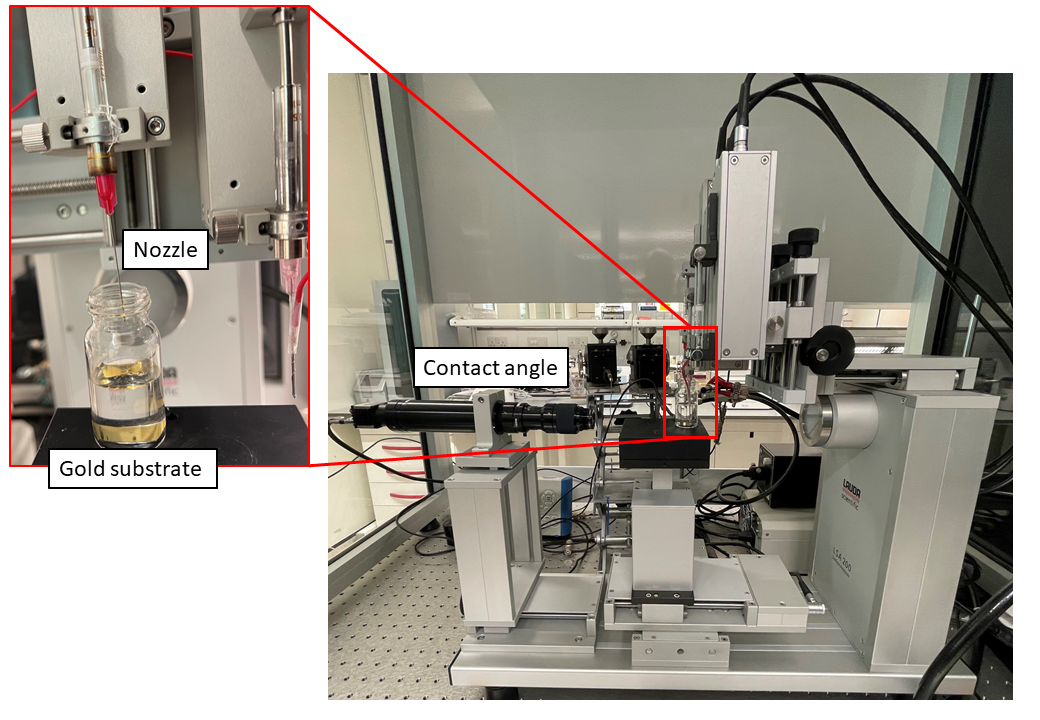

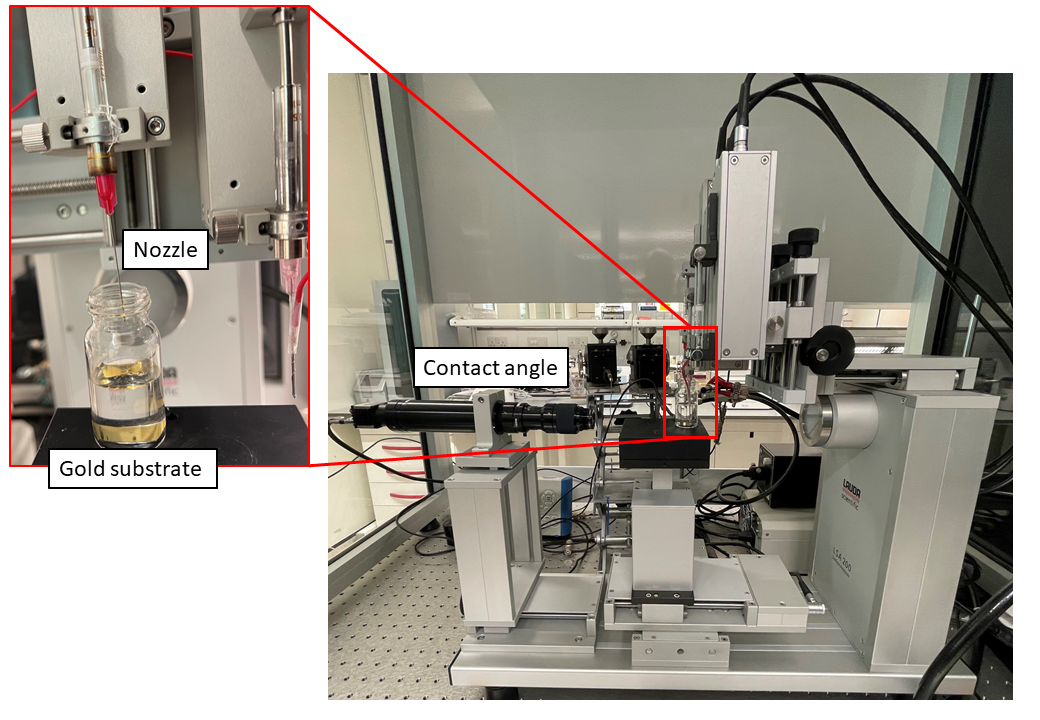


Figure S1. Experimental setup for creating molecular building-block-based 3D structures consisting of contact angle system, a nozzle for the injection of the molecules. Images and videos were captured with resolution of 1920 x 1200 px, and 170 fps.

**Experimental results**
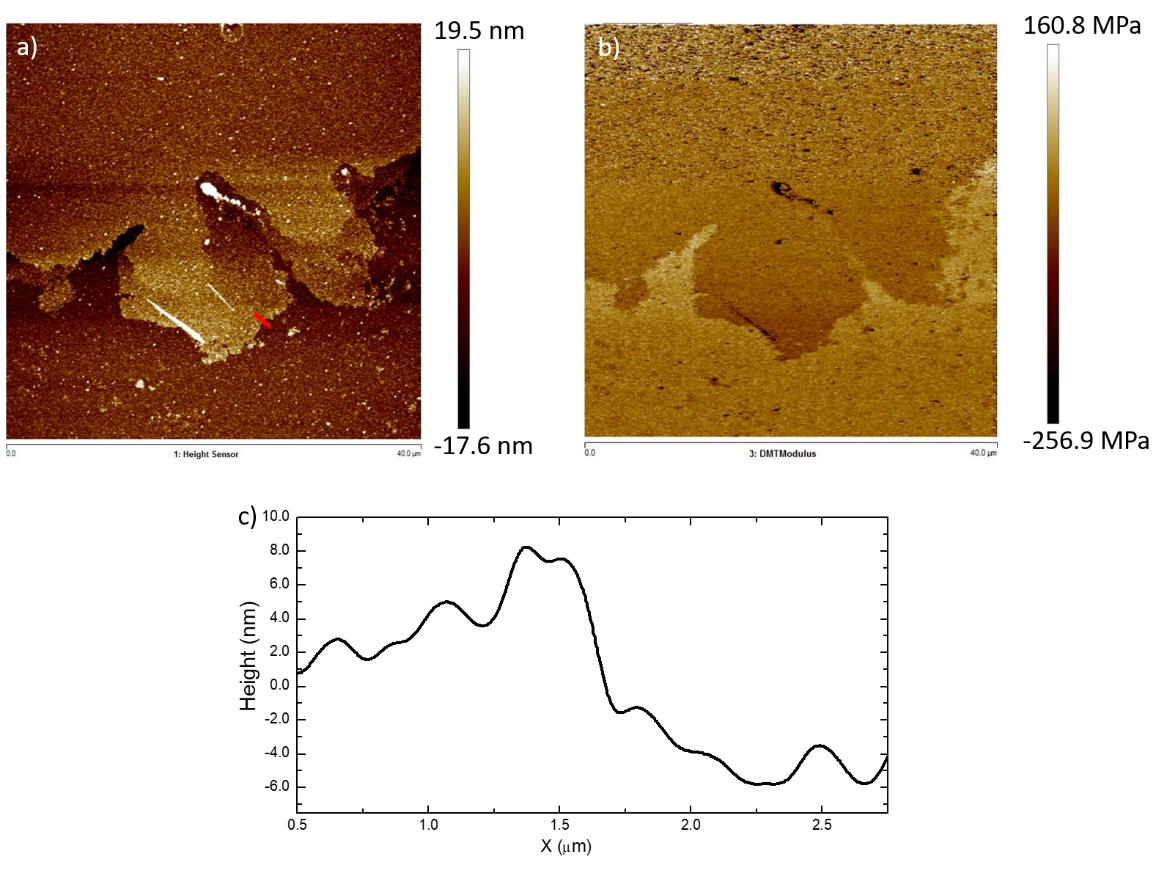


Figure S2: a) AFM images of the 3D structures created using n-hexane: a) the height AFM image, b) the DMT module mapping image and c) an interface section between one membrane layer and glass substrate showing a thickness average of around 5 nm.


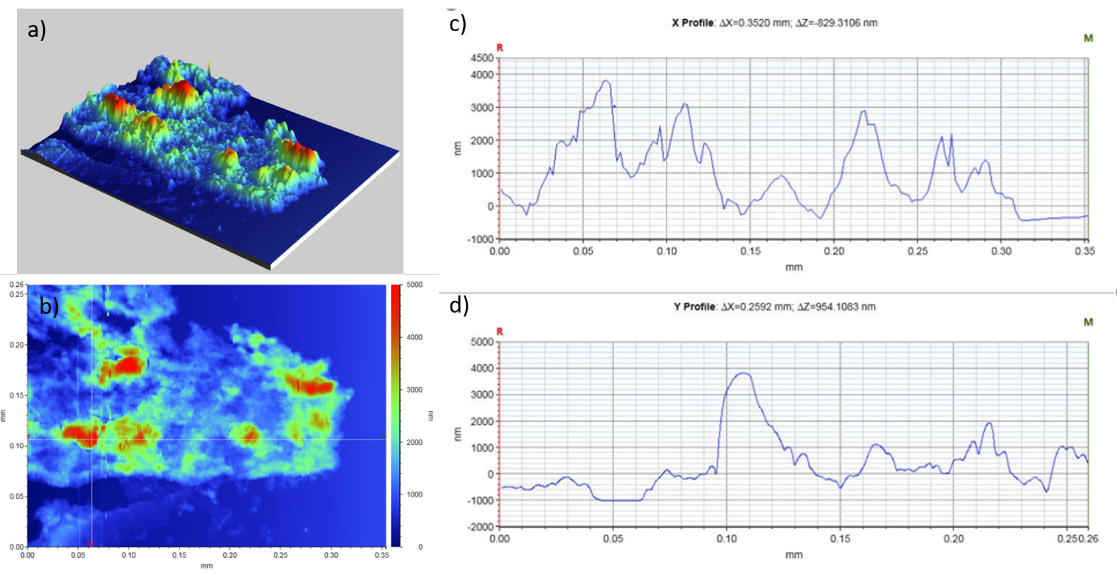


Figure S3: (a) 3D and (b) 2D profilometer image showing the thickness of the 3D structure created using ethanol. (c, d) Thickness variations along x- (c) and y- (d) directions.


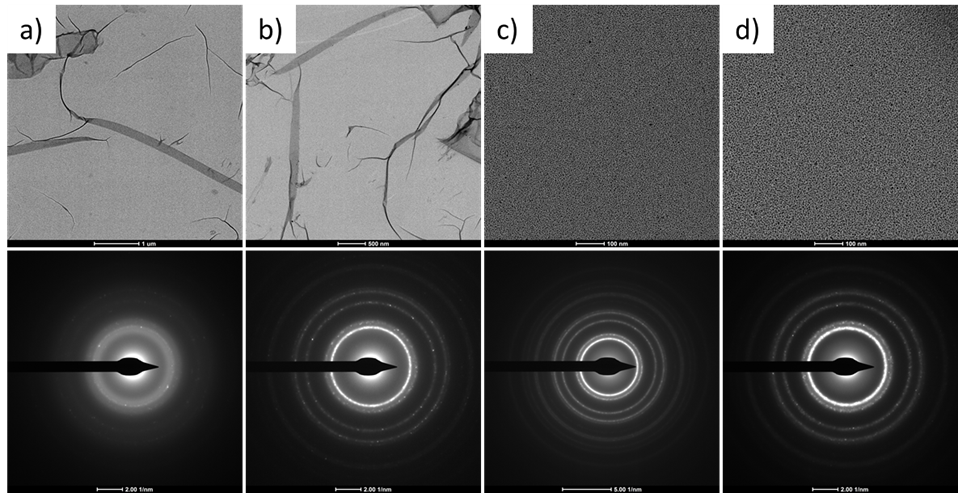


Figure S4: Transmission electron microscopy (TEM) images recorded for the thin film after annealing at following temperatures**: (a) 100** °C**, (b)** 200 °C, (c) 400 °C, (d) 550 °C. The formation of crystalline silver nanoparticles was confirmed by the selected area electron diffraction (SAED) pattern at each temperature.


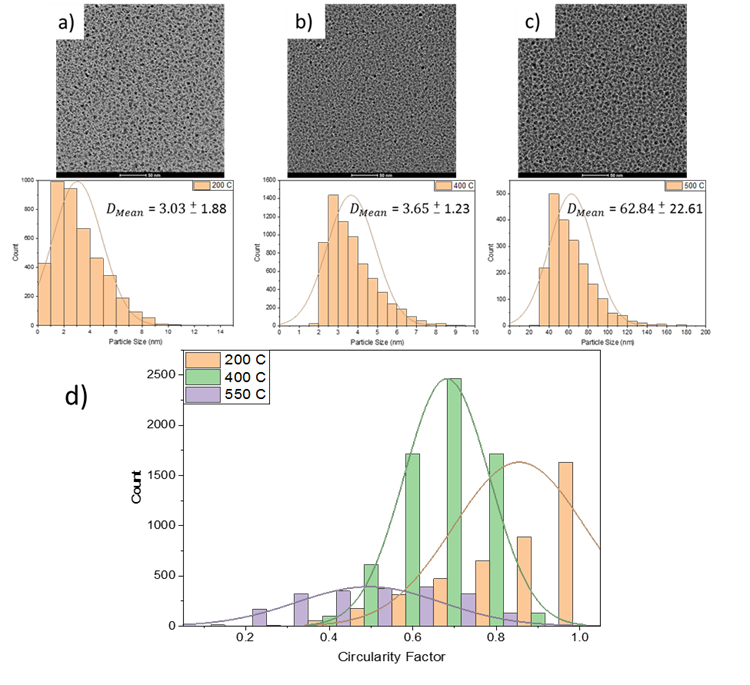


Figure S5: Particles size distribution of Ag nanoparticles in different annealing temperatures: a) 200 °C, b) 400 °C and c) 550 °C. d) The circularity factor of the nanoparticles at different annealing temperatures.


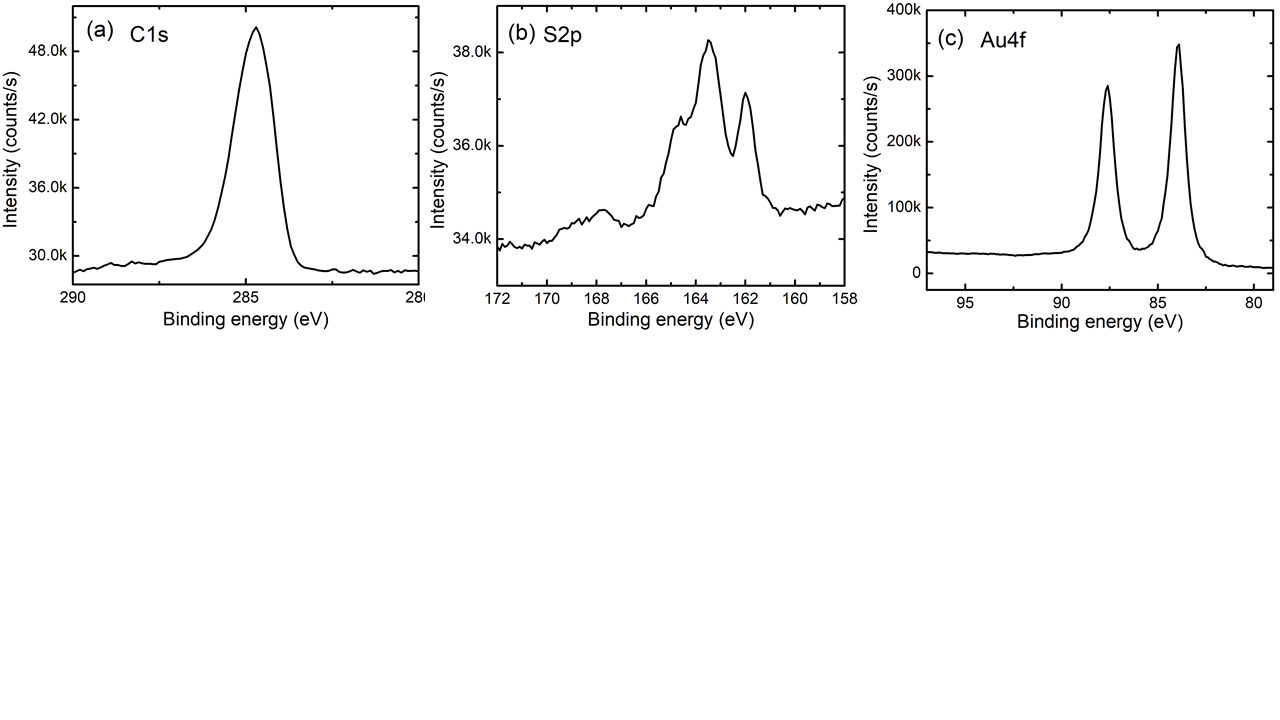


**Figure S6.** **XPS chemical analysis.** C1s (a), S2p (b) and Au4f (c) core level XPS spectra of self-assembled monolayer of C9 dithiol molecules on Au (111) surface.

**Movie captions**

Caption for Movie 1: Formation processes of 2D nanomembrane using n-hexane as a solvent for C9 molecules.

Caption for Movie 2: Qualitative demonstration of mechanical robustness of the 3D printed structures.

Caption for Movie 3: Formation processes of 3D carbon structure using ethanol as a solvent for C9 molecules.

Caption for Movie 4: Absence of self-heling when two separately printed structure (using n-hexane) are brought together.

Caption for Movie 5: Demonstration of self-healing when the second object is brought in contact with the first object with continuous injection of the molecules.

Caption for Movie 6: Self-healing process of two tubular structures to form a single tubular sample.

Caption for Movie 7: Demonstration of large-scale 3D printing of metal-organic hybrid structure (the video was accelerated 16 times).
